# Supplementary material for: Patterns of rates of mortality in the Clinical Practice Research Datalink
Source: PLoS One. 2022 Aug 4;17(8):e0265709. doi: 10.1371/journal.pone.0265709 (PMC9352072; doi:10.1371/journal.pone.0265709)
Supplement: S1 File — (DOCX) [file pone.0265709.s001.docx]

**Supplemental Material**

**Table S1. SMR by calendar year for sub-cohorts with minimum lookback window requirement**

|  | **Standardised mortality ratio (SMR) for lookback window period w cohort; overall and by calendar year** | | | | |
| --- | --- | --- | --- | --- | --- |
|  | **0** | **1** | **2** | **5** | **10** |
| Overall SMR | 0.980 (0.973-0.987) | 0.905 (0.898-0.912) | 0.881 (0.874-0.888) | 0.849 (0.841-0.857) | 0.837 (0.827-0.847) |
| Calendar year |  |  |  |  |  |
| *2000* | 0.956 (0.927-0.986) | 0.903 (0.937-0.870) | 0.875 (0.839-0.913) | 0.842 (0.797-0.890) | 0.861 (0.793-0.935) |
| *2001* | 0.970 (0.942-0.999) | 0.912 (0.943-0.883) | 0.877 (0.844-0.911) | 0.849 (0.807-0.893) | 0.830 (0.772-0.892) |
| *2002* | 0.985 (0.958-1.013) | 0.920 (0.949-0.892) | 0.898 (0.868-0.928) | 0.882 (0.842-0.924) | 0.871 (0.816-0.930) |
| *2003* | 1.001 (0.974-1.029) | 0.933 (0.961-0.906) | 0.906 (0.877-0.935) | 0.876 (0.838-0.915) | 0.836 (0.786-0.890) |
| *2004* | 1.027 (1.000-1.055) | 0.954 (0.982-0.927) | 0.927 (0.900-0.956) | 0.913 (0.877-0.949) | 0.920 (0.868-0.975) |
| *2005* | 0.977 (0.951-1.005) | 0.908 (0.936-0.882) | 0.892 (0.865-0.920) | 0.857 (0.826-0.889) | 0.833 (0.784-0.885) |
| *2006* | 0.973 (0.946-1.000) | 0.891 (0.918-0.865) | 0.867 (0.841-0.894) | 0.851 (0.822-0.882) | 0.851 (0.805-0.900) |
| *2007* | 0.963 (0.937-0.990) | 0.899 (0.925-0.873) | 0.875 (0.849-0.902) | 0.839 (0.811-0.868) | 0.831 (0.789-0.876) |
| *2008* | 0.968 (0.941-0.995) | 0.900 (0.927-0.874) | 0.877 (0.851-0.904) | 0.847 (0.819-0.876) | 0.853 (0.812-0.897) |
| *2009* | 0.946 (0.920-0.973) | 0.869 (0.895-0.843) | 0.854 (0.828-0.881) | 0.831 (0.804-0.860) | 0.818 (0.782-0.857) |
| *2010* | 0.998 (0.970-1.026) | 0.925 (0.953-0.898) | 0.902 (0.874-0.930) | 0.881 (0.852-0.911) | 0.870 (0.835-0.906) |
| *2011* | 0.982 (0.954-1.010) | 0.897 (0.925-0.870) | 0.875 (0.847-0.904) | 0.836 (0.808-0.866) | 0.827 (0.793-0.861) |
| *2012* | 0.991 (0.962-1.020) | 0.919 (0.948-0.891) | 0.891 (0.863-0.920) | 0.856 (0.827-0.886) | 0.850 (0.817-0.883) |
| *2013* | 0.996 (0.966-1.026) | 0.905 (0.935-0.876) | 0.881 (0.852-0.911) | 0.836 (0.806-0.868) | 0.827 (0.794-0.861) |
| *2014* | 0.950 (0.919-0.982) | 0.870 (0.902-0.840) | 0.844 (0.813-0.876) | 0.811 (0.780-0.844) | 0.798 (0.764-0.834) |
| *2015* | 1.002 (0.966-1.039) | 0.897 (0.933-0.862) | 0.877 (0.842-0.913) | 0.846 (0.810-0.884) | 0.842 (0.803-0.883) |
| *2016* | 0.915 (0.874-0.958) | 0.824 (0.866-0.785) | 0.805 (0.765-0.847) | 0.767 (0.726-0.811) | 0.764 (0.719-0.810) |
| *2017* | 0.974 (0.925-1.026) | 0.875 (0.925-0.827) | 0.853 (0.804-0.904) | 0.824 (0.774-0.877) | 0.820 (0.767-0.876) |
| *2018* | 1.045 (0.990-1.104) | 0.943 (1.000-0.889) | 0.921 (0.866-0.979) | 0.881 (0.825-0.941) | 0.838 (0.780-0.902) |

**Table S2. SMR by time-since-entry follow-up period for the unrestricted 1 million CPRD GOLD ransom sample**

| Standardised mortality ratio (SMR) by follow-up period | |
| --- | --- |
|  |  |
| 0-1 | 1.571 (1.545-1.597) |
| 1-2 | 1.095 (1.072-1.119) |
| 2-3 | 0.998 (0.975-1.022) |
| 3-4 | 0.971 (0.948-0.996) |
| 4-5 | 0.933 (0.908-0.958) |
| 5-6 | 0.877 (0.852-0.902) |
| 6-7 | 0.872 (0.847-0.898) |
| 7-8 | 0.843 (0.818-0.869) |
| 8-9 | 0.845 (0.818-0.872) |
| 9-10 | 0.813 (0.786-0.841) |
| 10-11 | 0.873 (0.844-0.903) |
| 11-12 | 0.830 (0.800-0.861) |
| 12-13 | 0.853 (0.821-0.886) |
| 13-14 | 0.820 (0.786-0.855) |
| 14-15 | 0.789 (0.753-0.827) |
| 15-16 | 0.823 (0.780-0.868) |
| 16-17 | 0.740 (0.691-0.794) |
| 17-18 | 0.795 (0.731-0.864) |
| 18-19 | 0.821 (0.746-0.902) |

**One million CPRD GOLD random sample**

A random sample of 1 million patients, aged 18 years or older with data linkages to both HES and ONS, alive with CPRD follow-up after 1 January 2000 was obtained from CPRD GOLD. The details are as follows:

An extract of all patients with research acceptable CPRD GOLD data was merged with practice level information (n= 19 483 855). The linkage eligibility of patients was further merged to the resultant file, providing details on an individual patient’s data linkage to both HES and ONS (n= 21 003 798). Those with no linkage information contained in the linkage eligibility file were removed (-11 559 592, n =9 444 206) along with those with linkage information (subjects had linkage eligibility information within the linkage file) but no data linkage to HES or ONS (-1 113 889, n= 8 330 317). Those without defined genders were removed (-84, n= 8 330 233).

Patients whose start date (latest of the date of first registration, current registration or practice data quality date) was after the administrative censoring date (31/12/2018) were removed (-255 572, n= 8 074 661). Those whose end date (earliest of the date of practice last data collection, transfer out, death or administrative censoring) was before the start of study (01/01/2000) were further removed (-635 208, n= 7 439 453). Patients whose start date was after their end date were removed (-542 473, n= 6 896 980) and finally, 1 376 287 patients whose index date (maximum of start date, 1^st^ Jan 2000 or 18^th^ birthday) was after their end date were removed, resulting in a final population of 5 520 693 patients from which a simple random sample without replacement of 1 million was drawn, Figure S1.


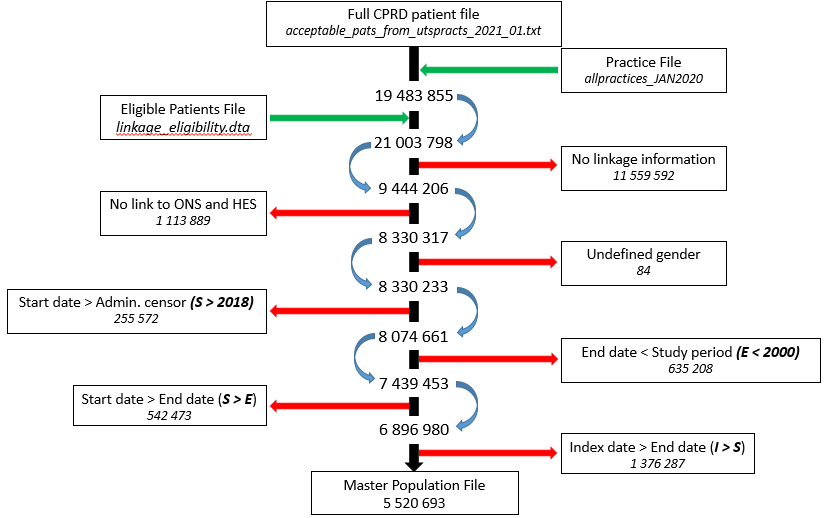


Figure S1. Generation of the ‘master population file’ from which the random sample of one million CPRD GOLD patients were drawn.

**Stata code for random sample of one million CPRD GOLD patients**

*Code to create patient ID file of randomly sample patients who are:

***research acceptable

***ONS & HES linked

***start date before Dec 2018 & end after Jan 2000 + start before end date

***be 18 or over at index

*=-=-=-=-=-=-=-=-=-=-=-=-=-=-=-=-=-=-=-=

*Data imports, conversions and linkages

clear

cls

*set directory

cd "~ /amendment data request"

*import practice file and save as Stata data file

import delimited "allpractices_JAN2021.txt", clear

save "allpractices_JAN2021", replace

*import linkage eligibility file and save as Stata data file

import delimited "~ \linkage_eligibility_new_patids.txt", clear

save "linkage_eligibility_new_patids", replace

*import research acceptable patients txt file

import delimited "acceptable_pats_from_utspracts_2021_01.txt", clear

count

*n = 19,483,855

*-------------------

*create practice ID from last 3 digits of patient ID

tostring patid, replace format(%12.0f)

gen pracid = substr(patid,-3,.)

destring patid, replace

destring pracid, replace

replace pracid = pracid + 10000

*merge patient and practice file

merge m:1 pracid using "allpractices_JAN2021.dta", nogen

count

*n = 19,483,855

*-------------------

*merge linkage eligibility file onto patient-practice file

merge 1:1 patid using "linkage_eligibility_new_patids.dta"

count

*n = 21,003,798

*=-=-=-=-=-=-=-=-=-=-=-=-=-=-=-=-=-=-=-=-=-=-=-=-=

*Remove subjects with no linkage and linkage info

*drop those with no linkage information

drop if _merge!=3

*11,559,592 dropped

count

*n = 9,444,206

*-------------------

*drop those with no link to HES or ONS

drop if hes_e!=1

drop if death_e!=1

drop _merge

count

*1,113,889 dropped

*n = 8,330,317

*=-=-=-=-=-=-=-=-=-=-=

*Change date formats

*date of birth

tostring yob, gen(yob_str)

gen dob_str = "01/07/" + yob_str

gen dob = date(dob_str, "DMY")

drop yob_str dob_str

*first registered date

gen first_reg = date(frd,"DMY")

*current register date*

gen cur_reg = date(crd,"DMY")

*reached quality

gen reach_qual = date(uts,"DMY")

*last collection date

gen last_col = date(lcd,"DMY")

*transfer-out date

gen trans_out = date(tod,"DMY")

*censoring date

gen censor = date("31/12/2018","DMY")

*CPRD death date

gen deaddate_CPRD = date(deathdate,"DMY")

*start date

egen start_date = rowmax(first_reg cur_reg reach_qual)

*end date

egen end_date = rowmin(last_col trans_out deaddate_CPRD censor)

*index date

*max(start date + 10 years, 01/01/2000, date trun 18)

gen start_d = date("01/01/2000","DMY")

gen eighteen_d = dob + 18*365.25

egen index_date = rowmax(start_date start_d eighteen_d)

drop start_d eighteen_d

*tidy up

drop frd crd uts lcd tod deathdate

format dob first_reg cur_reg reach_qual last_col trans_out deaddate_CPRD censor start_date end_date index_date %td

*region mapping

merge m:1 region using "~/region.dta"

drop if _merge!=3

drop _merge

*transfer out mapping

merge m:1 toreason using "~/transfer.dta"

drop if _merge!=3

drop _merge

count

* n = 8,330,317

*-------------------

*CPRD death indicator

gen dead_CPRD = 0

replace dead_CPRD = 1 if !missing(deaddate_CPRD)

*Death in follow-up period indicator

gen dead_cens = 0

replace dead_cens = 1 if dead_CPRD == 1 & deaddate_CPRD <= end_date

count

*n = 8,330,317

*=-=-=-=-=-=-=-=-=

*Remove subjects

*drop if gender unknown

drop if gender == 3

*84 removed

count

*n = 8,330,233

*-------------------

*drop if start date after 31 Dec 2018

drop if start_date > censor

*255,572 dropped

count

*n = 8,074,661

*-------------------

*drop if end date before 01 Jan 2000

drop if end_date < 14610

*635,208 droppped

count

*n = 7,439,453

*-------------------

*drop if start date after end date

drop if start_date > end_date

*542,473 dropped

count

*n = 6,896,980

*-------------------

*subset of patients with start date before Dec 2018, end dates after Jan 2000 & end dates after start dates

*include only those with index dates before their end date

keep if index_date < end_date

*1,376,287

count

*n = 5,520,693

*-=-=-=-=-=-=-=-=-=-=-=-=-=-=-=-=-=-=-=-=-=-=-=-

* Take random sample without replacement of 1m

set seed 123456

sample 1000000, count

*4,520,693 deleted

count

*n = 1,000,000

*-------------------

*save CPRD dataset

keep patid pracid region region_desc gender yob dob trans_out tor_desc first_reg cur_reg reach_qual last_col start_date index_date end_date deaddate_CPRD dead_cens

order patid pracid region region_desc gender yob dob first_reg cur_reg reach_qual last_col trans_out tor_desc deaddate_CPRD start_date index_date end_date dead_cens

save "~\1m_sample.dta", replace
